# Photothermal Cavitation-Driven Micromotor to Penetrate Cell Membrane

**DOI:** 10.1021/jacs.5c00482

**Published:** 2025-02-27

**Authors:** Binglin Zeng, Jialin Lai, Jingyuan Chen, Yaxin Huang, Qingxin Guo, Chao Huang, Xiaofeng Li, Changjin Wu, Shuai Li, Jinyao Tang

**Affiliations:** † Department of Chemistry, The University of Hong Kong, Kowloon, Hong Kong 999077, China; ‡ HKU-CAS Joint Laboratory on New Materials and Department of Chemistry, The University of Hong Kong, Kowloon, Hong Kong 999077, China; § Department of Mechanical Engineering, The University of Hong Kong, Kowloon, Hong Kong 999077, China; ∥ College of Shipbuilding Engineering, Harbin Engineering University, Harbin 150001, China; ⊥ State Key Laboratory of Synthetic Chemistry, The University of Hong Kong, Kowloon, Hong Kong 999077, China; ∇ Materials Innovation Institute for Life Sciences and Energy (MILES), HKU-SIRI, Shenzhen 518000, China

## Abstract

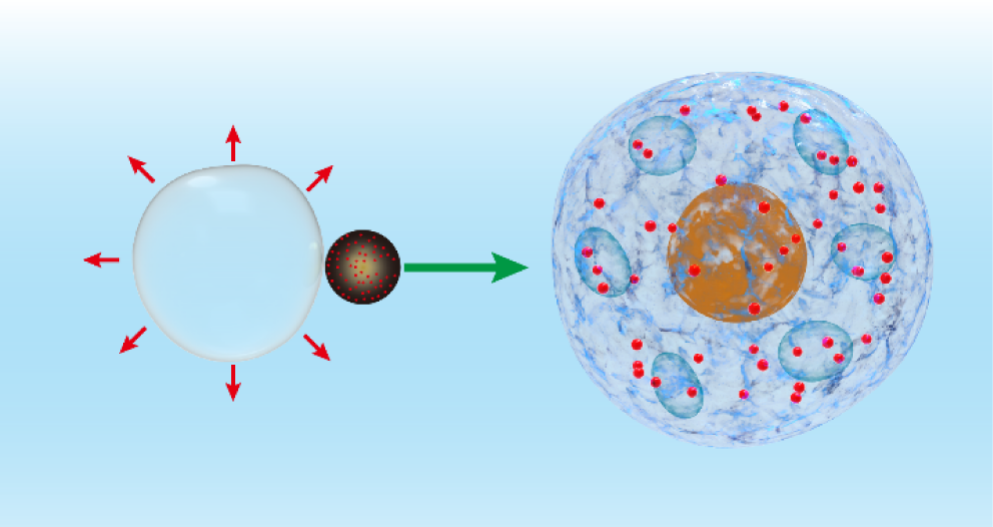

Photothermally driven micro/nanomotors efficiently convert light into mechanical motion, making them highly attractive for biomedical applications due to their exceptional biocompatibility and safety. However, one mystery of the photothermally driven micro/nanomotor is the wide range of reported light intensities applied, ranging from 1 W cm^–2^ to over 10^5^ W cm^–2^. To address this mystery, we systematically investigated the propulsion of a carbon microbottle-based micromotor under three illumination conditions: continuous laser, pulsed laser, and scanning laser, where a new cavitation-driven mechanism is identified. Using a high-speed camera, we find that the instantaneous deposition of laser energy on the micromotors can lead to transient and localized evaporation of the solvent, creating cavitation bubbles to drive micromotors with ultrafast speed, where instantaneous velocity over 1 m s^–1^ is observed. Through precise modulation of the scanning orientation and intensity of the laser, directional propulsion and targeted explosions of the microbottles are achieved, where the instant force is strong enough to penetrate live cell membranes. Finally, the cavitation-driven micromotors are exploited as gene transfection tools, where targeted cytoplasmic transfection is demonstrated.

## Introduction

Micro/nanomotors (MNMs), which can effectively convert ambient energy into mechanical propulsion, are being developed into viable biomedical tools for noninvasive targeted drug delivery and micromanipulation.^1,2^ Usually, the self-propulsion of MNMs is achieved through mechanisms such as self-diffusiophoresis,^3,4^ self-electrophoresis,^5,6^ and bubble-propelling,^7−9^ where the chemical reaction on the MNM surface is required. On the other hand, the external field-propelled motors are powered with direct external energy input, such as magnetic,^10^ electric,^11^ acoustic,^12−14^ light,^15^ or their combinations,^16,17^ promising improved biocompatibility and bioavailability. The autonomously untethered motions of these MNMs offer promising applications in diverse fields such as environmental remediation,^18^ sensing,^19^ micromolding,^20^ and biomedicine.^21^

Particularly, in the application of biomedicine, MNMs are anticipated to navigate noninvasively through the human body, reaching disease-affected regions inaccessible to traditional medical devices.^22,23^ They are expected to perform specialized tasks, including targeted drug delivery,^1,24^ noninvasive surgery,^25^ precise nanosurgery,^26^ and detoxification.^27^ However, the practical biomedical uses are still limited in spite of the great advancements in micromotors over the past decades. Currently, only a limited propulsion mechanism has been adopted for biomedical MNM applications, which is mainly focused on magnetic field-driven and biohybrid MNMs,^28,29^ while few demonstrate sufficient driving force to overcome physiological barriers, such as the blood-brain barrier, blood-testis barrier, mucus, and cell membrane barrier.^23,30^

On the other hand, extensive research has focused on developing optically controlled micro/nanomotors due to the advantages of control flexibility and biocompatibility for therapeutic and diagnostic procedures in biomedicine.^31^ Initially, it has been demonstrated that a continuous defocused laser beam can propel Janus particles through self-thermophoresis, with the laser intensity of ∼10^5^ W cm^–2^.^32^ Our recent micro/nanomotor study also indicated that similar light intensity would be required to generate a sufficient temperature gradient even with surface optimization.^33^ In contrast to a continuous laser, both scanning and pulsed lasers have been demonstrated to effectively propel micromotors at significantly lower laser intensities in the order of W cm^–2^,^31,34,35^ indicating that a drastic power reduction can be achieved by concentrating continuous photon energy into short pulses with orders of magnitude higher than instant power.^35,36^ Once instantaneously depositing huge amounts of energy onto micromotors, various effects may come into play, such as the Soret effect^37^ or natural convection,^38^ resulting from temperature gradients or cavitation arising from thermotropic phase transition.^39^ However, the role of such effects in propelling MNMs through scanning or pulsed lasers is largely missing.

In this study, the carbon-based microbottle (CMB) is prepared as a micromotor model, and its motion modes under a continuous laser, a nanosecond pulsed laser, and a scanning laser are systematically investigated. We discovered that nanosecond pulsed and scanning lasers efficiently propel CMBs at significantly lower light intensities of approximately 0.05 W cm^–2^ and 10 W cm^–2^, respectively, compared to continuous global laser illumination, which requires around 3000 W cm^–2^. The inconsistency in the laser intensity for propelling CMBs using these three types of lasers is thoroughly investigated. Surprisingly, the nanosecond pulsed and scanning lasers can propel CMBs with ultrahigh velocities exceeding 1 m s^–1^ and 100 μm s^–1^, respectively. The underlying mechanisms behind these ultrafast motions are revealed through numerical simulations and an experimental analysis. In contrast to the prevailing accepted self-thermophoresis mechanism, the pulsed and scanning lasers propel the micromotors by generating transient cavitation microbubbles, which create propulsion forces far greater than thermophoretic flow. Furthermore, we demonstrate the potential of scanning laser-propelled CMBs for targeted cell navigation and their ability to penetrate membrane barriers through cavitation bubbles, thereby enhancing the plasmid transfection efficiency. These findings provide valuable insights into the field of laser-propelled micromotors and highlight their potential to revolutionize various biomedical procedures.

## Results and Discussion

### Light-Intensity-Dependent Motion Behaviors

**Figure 1:**
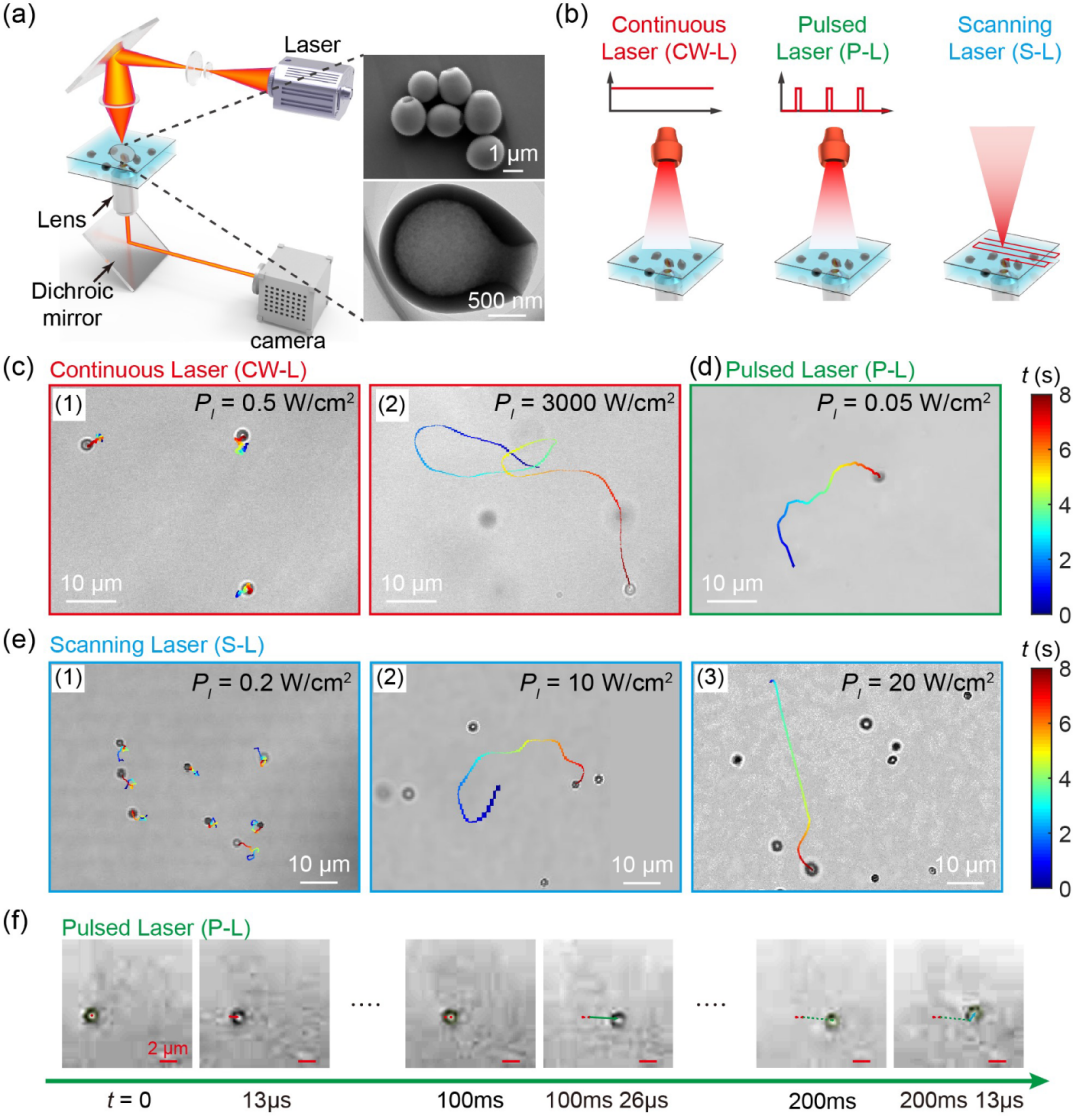
Motion modes of photothermal-driven CMBs. (a) Schematic of the laser setup for CMB propulsion. The inset is the SEM and TEM images of the as-prepared CMB swimmers. (b) Three laser conditions: continuous, defocused pulsed, and scanning lasers. (c) Trajectories of CMBs under a continuous laser with intensities of 0.5 and 3000 W cm^–2^. (d) Trajectory of a CMB under a defocused pulsed laser with an intensity of 0.05 W cm^–2^. (e) Trajectories of CMBs under a scanning laser with intensities of 0.2, 10, and 20 W cm^–2^. (f) High frame rate optical images of a CMB under a defocused pulsed laser with an intensity of 0.05 W cm^–2^.

**Figure 2:**
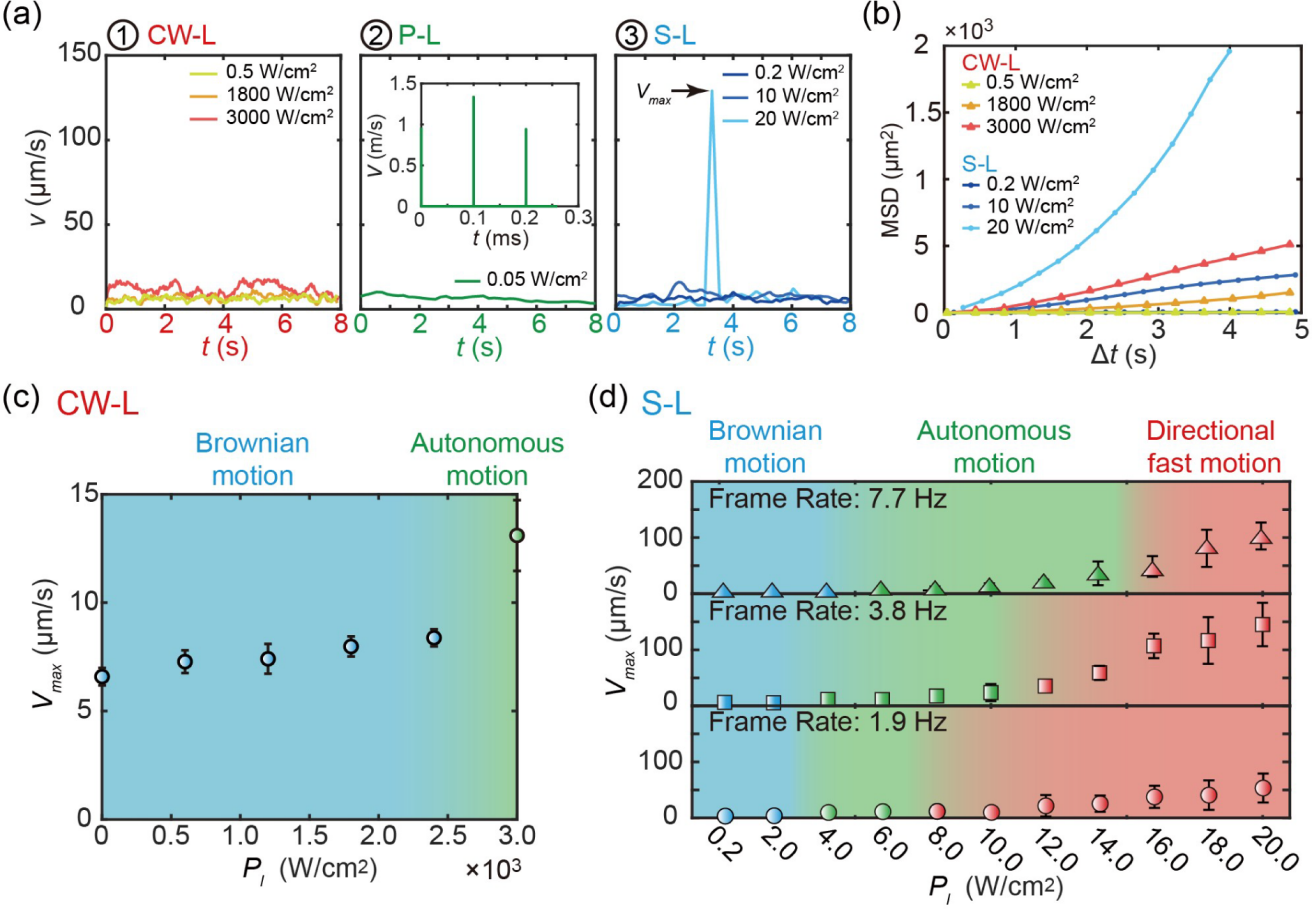
Characterization of propulsion velocity of CMBs. (a) Instantaneous velocity of CMBs under a continuous laser at intensities of 0.5, 1800, and 3000 W cm^–2^, a defocused pulsed laser at an intensity of 0.05 W cm^–2^, as well as a scanning laser at intensities of 0.2, 10, and 20 W cm^–2^. (b) Mean square displacement (MSD) of CMBs under the scanning and continuous lasers for various intensities. (c, d) Phase diagram of the maximum velocity of CMBs under the continuous laser (c) and the scanning laser (d).

The propulsion of CMBs in water was monitored using an optical camera under different laser conditions: continuous, defocused pulsed, and scanning lasers (Figure 1a,b). Figure 1c shows typical moving trajectories of CMBs under a continuous laser. Under an intensity of 0.5 W cm^–2^, CMBs exhibit Brownian motion (Figure 1c-1) due to thermal fluctuation. However, when exposed to a laser intensity of 3000 W cm^–2^, CMBs demonstrated nondirectional autonomous motion (Figure 1c-2 and Movie S1), with an average velocity of approximately 10 μm s^–1^ (Figure 2a-1).

**Figure 3:**
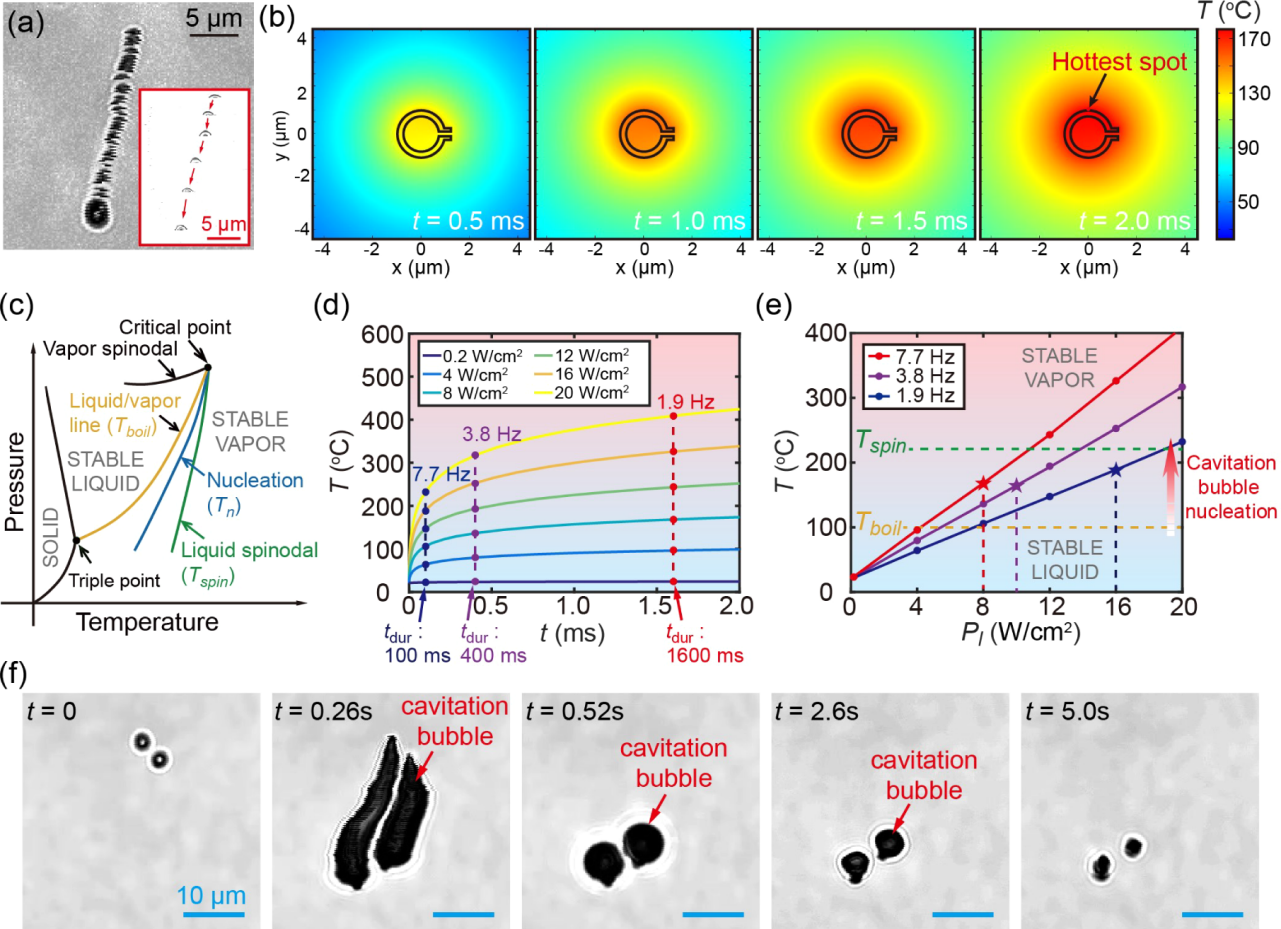
Photothermal effect induced temperature increase on CMBs. (a) Zoomed-in optical image of a CMB with directional motion at a scanning speed of 1000 line s^–1^. The inset illustrates the same phenomenon at a scanning speed of 100 line s^–1^. (b) Numerical temperature field around a CMB under laser scanning through a CMB at an average laser power of 8 W cm^–2^. (c) Schematics of the phase diagram of water. (d) Evolution of hottest spot temperature on a CMB in time for various laser intensities. (e) Hottest spot temperature on a CMB as a function of laser intensity with scanning rates of 7.7, 3.8, and 1.9 Hz. (f) Successive images of CMBs propelled by cavitation bubbles in hyaluronic acid hydrogel.

In contrast to a continuous laser, defocused pulsed and scanning lasers can propel CMBs at significantly lower light intensities. The defocused pulsed laser, with an intensity as low as 0.05 W cm^–2^ (Figure 1d and Movie S2), and the scanning laser, with an intensity of 10 W cm^–2^ (Figures 1e-2, Movie S3), were found sufficient to overcome thermal fluctuations (Figure 1e-1, Movie S4) for CMB propulsion. Moreover, when the scanning laser intensity was increased to 20 W cm^–2^, CMBs exhibited rapid shooting behaviors (Figure 1e-3 and Movie S5), where their motion direction could be modulated by the scanning orientation (Movie S6). High-speed camera monitoring of CMBs under the defocused pulsed laser revealed a similar rapid shooting behavior (Figure 1f and Movie S7).

The ability of defocused pulsed and scanning lasers to propel CMBs at lower laser intensities is attributed to their high instantaneous intensity compared to that of the continuous laser. In the case of a continuous laser, where the laser energy was continuously deposited on the CMBs, there was an equivalence between instantaneous and average light intensity. However, in the cases of defocused pulsed and scanning lasers, laser energy deposition occurred over a pulse or scanning spot, resulting in instantaneous light intensity deposition on the CMBs that might be several orders of magnitude higher than the average value obtained from a light intensity meter. For example, the employed defocused pulsed laser had a dwell time of *t*_p_ = 6 ns and operated at a frame rate of *f*_r_ = 10 Hz, while maintaining an average laser intensity of  = 0.05 W cm^–2^. By calculating the instantaneous laser intensity as *P*_ins_ = /(*f*_r_ × *t*_p_), we determined that the value of *P*_ins_ was approximately 10^4^ W cm^–2^. Furthermore, the scanning laser operating at an average intensity of 20 W cm^–2^ and a scanning rate of 7.7 Hz, resulted in an approximate instantaneous light deposition of 26 000 W cm^–2^ on a CMB (see Supporting Information S2.2).

The instantaneous energy deposition on CMBs was also evident from the evolution of the propulsion velocity *v* of CMBs. As illustrated in Figure 2a, the instantaneous velocities of CMBs under defocused pulsed laser irradiation with  = 0.05 W cm^–2^ and scanning laser irradiation with  = 20 W cm^–2^ can reach 10^6^ μm s^–1^ and 130 μm s^–1^, respectively, which are significantly higher than the average velocity (*v* ≈ 10 μm s^–1^) observed during the autonomous motion of CMBs. Moreover, the mean square displacement (MSD) shown in Figure 2b indicated that the shooting motion of CMBs under scanning laser irradiation ( = 20 W cm^–2^) corresponds to directed motion and superdiffusion.

To gain more insight into the motion modes of CMBs, we conducted a quantitative investigation of their maximum propulsion velocity under continuous and scanning lasers. As illustrated in Figure 2c, the motion modes of CMBs under a continuous laser depend on the light intensity. When exposed to a continuous laser with intensities ranging from 0.5 to 2500 W cm^–2^, thermal fluctuations primarily dictated the motion of CMBs (Figure 1c-1), resulting in maximum velocities between 6 and 8 μm s^–1^ (Figure 2c). At a laser intensity of 3000 W cm^–2^, the propulsion force enabled CMBs to effectively overcome thermal fluctuations, demonstrating a clear autonomous motion with a maximum velocity of approximately 13 μm s^–1^.

In contrast to the propulsion of CMBs under a continuous laser, the motion modes of CMBs driven by the scanning laser were governed by not only the laser intensity but also the scanning rate. As depicted in Figure 2d, with laser intensities ranging from 0 to 20 W cm^–2^ and scanning rates of 7.7, 3.8, and 1.9 Hz, three distinct types (Figure 1e) of motion behavior for CMBs could be classified. At a scanning rate of 7.7 Hz, light intensities between 0.4 and 4.0 W cm^–2^ resulted in Brownian movement, while intensities between 6.0 and 14.0 W cm^–2^ led to autonomous motion. For light intensities from 16.0 to 20.0 W cm^–2^, the CMBs exhibited directional shooting. At scanning rates of 3.8 and 1.9 Hz, the minimum laser power required to induce directional shooting decreases to 12 and 8 W cm^–2^, respectively (Figure 2d). The dependence of propulsion modes of CMBs on scanning frequency stems from the fact that a higher scanning frequency results in a shorter duration of laser beam contact with a CMB per scanning cycle, thereby reducing the energy deposition on the CMB. As a result, a higher light intensity is required to overcome thermal fluctuations and achieve effective propulsion of CMBs. Therefore, theoretically, if the scanning frequency is increased further, the energy deposition per scanning cycle may become insufficient to propel CMBs.

### Driving Mechanism of CMBs

The photothermal effect of carbon materials enables CMBs to achieve propulsion, effectively overcoming thermal fluctuations. By assessing the temperature increase in aqueous solutions containing CMBs at various concentrations,^40−42^ we evaluated the photothermal properties of CMBs (Figure S1), thereby demonstrating their excellent photothermal performance (Supporting Information S1.1). In the majority of photothermal-driven micromotors, whether propelled by continuous or scanning lasers, thermophoresis is widely acknowledged as the predominant and efficient propulsion mechanism.^32,43^ However, we note here that thermophoresis may play a partial role during the autonomous motion (marked by green in Figure 2d) of CMBs, but the thermophoresis effect can be neglected during CMBs’ directional and ultrafast motion process (marked by red in Figure 2d). Given that the asymmetric structure of the CMB originates from its open end, the temperature gradient across the motor is theoretically aligned from the head to the open end, resulting in a self-propulsion force parallel to the central axis of symmetry of a CMB. Consequently, thermophoresis should cause each CMB to move along its central axis of symmetry rather than producing a unified directional motion (Figure 1e-3 and Movie S6).

To further confirm that thermophoresis is not the dominant mechanism in this ultrafast propulsion process, we theoretically calculated the temperature gradients ∇*T* across a CMB under a light intensity of 3000 W cm^–2^. This results in ∇*T* ≈ 0.8 K μm^–1^, leading to a propulsion velocity of approximately 4.4 μm s^–1^. This suggests that thermophoresis may play only a partial role during autonomous motion, where observed velocities are around 10 μm s^–1^. However, the value of 4.4 μm s^–1^ is significantly lower than the observed velocities of 130 μm s^–1^ (Figure 2a-3) during the directional motion of CMBs. To achieve such high propulsion velocities, temperature gradients would need to be as high as 23 K μm^–1^, which is an order of magnitude higher than the typically observed values of less than 2 K μm^–1^ in photothermal micromotor systems^44,45^ (see Supporting Information S2.3).

In contrast to thermophoresis, thermotropic phase transitions are more likely to generate the significant propulsion velocities observed in Figure 2a-2,3. Given the photothermal conversion capability of carbon materials,^46^ laser irradiation can induce localized heating of the water around and inside CMBs, which may result in thermal expansion of water inside the CMB or water vaporization. We note that the thermal expansion of water plays a negligible role in CMB propulsion based on our simulation results. Specifically, the propulsive force generated by the thermal expansion of water within CMBs can not sustain their continuous propulsion at approximately 10 μm s^–1^ (Supporting Information S2.4) under a continuous laser with  of 3000 W cm^–2^ (Figure 2a-1). Additionally, an experiment involving the propulsion of CMBs in a thin film of Triton X-100 demonstrated that their movement was primarily driven by the generation of vapor bubbles (Movie S8). Thus, we speculated that the nucleation of vapor bubbles played an important role in laser-driven CMBs.

To further reveal the underlying mechanism of vapor bubble nucleation and the resultant directional shooting of CMBs under a scanning laser, a comprehensive characterization of this phenomenon was conducted. Figure 3a presents a zoomed-in optical image of a CMB exhibiting directional motion captured by a confocal microscope, with the inset highlighting this occurrence at a decreased laser scanning speed of 100 lines s^–1^. Notably, during the course of directional motion, a trailing effect was observed. Specifically, the inset in Figure 3a indicated that as the laser scanned across the upper region of the CMB, the CMB repeatedly moved downward until eventually exiting the imaging window. This indicates a significant propulsive force acting on the CMB when the scanning laser interacts with its top portion, corresponding to a localized phase transition of the surrounding water.

To verify the localized phase transition near the upper region of the CMB under a scanning laser, we conducted numerical simulations to estimate the temperature distribution in the vicinity of the CMB when the laser scanned over its upper portion (Supporting Information S2.5). Figure 3b displays the temperature field around a CMB under an average power of 8 W cm^–2^, corresponding to an instantaneous power of 15 600 W cm^–2^ over a duration of 2 ms. The highest recorded temperature was observed at the top point of the CMB, reaching 170 °C. The temperature increase might induce a phase transition in the surrounding medium. The phase diagram of water, depicted in Figure 3c, shows the maximum attainable temperature for liquid water under a given pressure, known as the spinodal temperature (represented by the green curve in Figure 3c). At this temperature, spontaneous homogeneous nucleation of vapor bubbles occurs. However, in most cases, bubble nucleation occurs at temperatures lower than the spinodal temperature due to the presence of microscopic cracks, cavities, or pits filled with gas, impurities, or clusters of gas molecules that act as nucleation centers.^47,48^ This process is termed heterogeneous nucleation (indicated by the blue curve in Figure 3c).

Figure 3d displays the temporal evolution of temperature at the hottest spot on the CMB for average laser intensities ranging from 0.2 to 20 W cm^–2^. Considering that the dwell time of the scanning laser on a CMB was not constant and depended on the scanning rate (Supporting Information S2.2), the duration *T*_dur_ of laser irradiation on the top portion of bottles can be estimated based on their size and the applied scanning rate. As illustrated in Figure 3d, *T*_dur_ values are 100, 400, and 1600 μs for scanning frame rates of 7.7, 3.8, and 1.9 Hz, respectively. The relationship between temperature *T* and laser power  for these three different scanning frame rates is depicted in Figure 3e, demonstrating a linear dependence between maximum temperature and laser power as it increases from 0.2 to 20 W cm^–2^.

As shown in Figure 2d, directional shooting of CMBs was induced at laser intensities () of 8, 10, and 16 W cm^–2^ for scanning frame rates of 7.7, 3.8, and 1.9 Hz, respectively. The corresponding theoretical temperatures for these scanning rates can be derived from the results in Figure 3e, represented by red, purple, and blue stars, respectively. By reference to the phase diagram in Figure 3c and considering the theoretical temperature values obtained, it was evident that the nucleation temperature associated with the phase transition falls within the range between the boiling temperature and the spinodal temperature regions. This observation was consistent with previous research findings.^49^

Given that the lifecycle of a cavitation bubble is on the microsecond time scale,^49^ which is considerably shorter than the employed minimum scanning cycle (∼130 ms) of the confocal microscope, it poses a significant challenge to capture cavitation events in water using this technique. To confirm the nucleation of cavitation bubbles, we conducted an experiment involving scanning-laser-driven CMBs within a hydrogel matrix comprising 0.22% hyaluronic acid and 0.11% agar. The utilization of this hydrogel effectively extended the lifespan of cavitation bubbles, enabling their visualization through confocal microscopy. As displayed in Figure 3f and Movie S9, the formation and subsequent collapse of microbubbles, approximately 5 μm in diameter, were observed on top of the CMBs during their directional shooting. This observation not only provided compelling evidence for the critical role of cavitation bubbles in governing the directional shooting exhibited by CMBs under the scanning laser but also demonstrated that these laser-driven CMBs can propel through highly viscous fluids.

### Interaction between a Cavitation Bubble and a Micromotor

A numerical simulation was conducted to further elucidate the mechanism by which cavitation bubbles induce directional shooting of a microparticle under a scanning laser. For simplification, a microparticle was used in place of a CMB. In this simulation, based on the experimental images (Figures 1a, 3f, and 6a), the particle diameter and maximum cavitation bubble diameter were set to 2 and 5 μm, respectively. In theory, the relative size ratio between the bubble and the particle determines whether the bubble detaches from the particle prior to collapse.^50^ Specifically, if the ratio of the bubble diameter to the particle diameter exceeds 2, as observed in this study, the bubble will detach before collapse.

**Figure 4:**
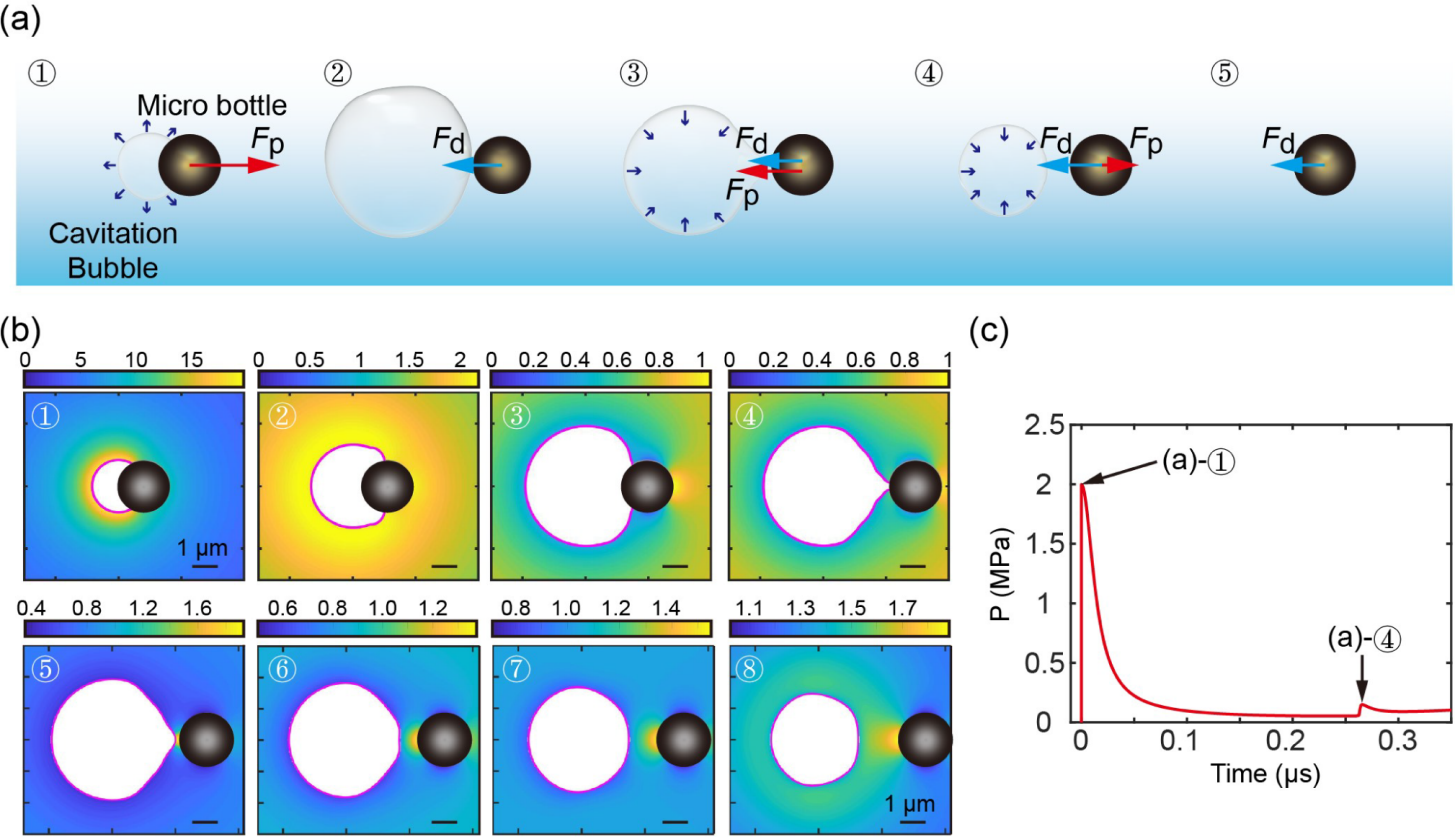
Numerical model of the interaction between a cavitation bubble and a CMB. (a) Sketch of the interaction between a cavitation bubble (diameter of 5 μm) and a CMB (diameter of 2 μm). *F*_p_ and *F*_d_ indicate the propulsion force and drag force, respectively. (b) Interaction and pressure contours in the numerical simulation. (c) Temporal evolution of the pressure acting on a CMB due to bubble cavitation.

Figure 4a,b displays the sketch and the pressure contours during the interaction between a cavitation bubble and a microparticle, respectively. The initial stages of bubble expansion, as indicated by the simulations, are shown in Figure 4b(1–3), while the subsequent collapse states are depicted in Figure 4b(4–8). Additionally, the time-dependent pressure intensity exerted on the CMB was extracted (Figure 4c). The pressure–time curves exhibit an initial peak value of approximately 2 MPa, indicating a propelling force *F*_p_ (Figure 4a-1) exerted on the CMB during the rapid growth of the bubble, followed by a secondary weaker peak during the disconnection between the bubble and the particle, corresponding to a relatively weak *F*_p_ (Figure 4a-4). Thus, as depicted in Figure 4a, the propulsion of the microparticle is primarily driven by the rapid expansion of the cavitation bubble, followed by a depletion of kinematic energy due to Stokes drag *F*_d_ after bubble collapse.

### Enhancement of CMB Propulsion by Introducing Nanobubbles

**Figure 5:**
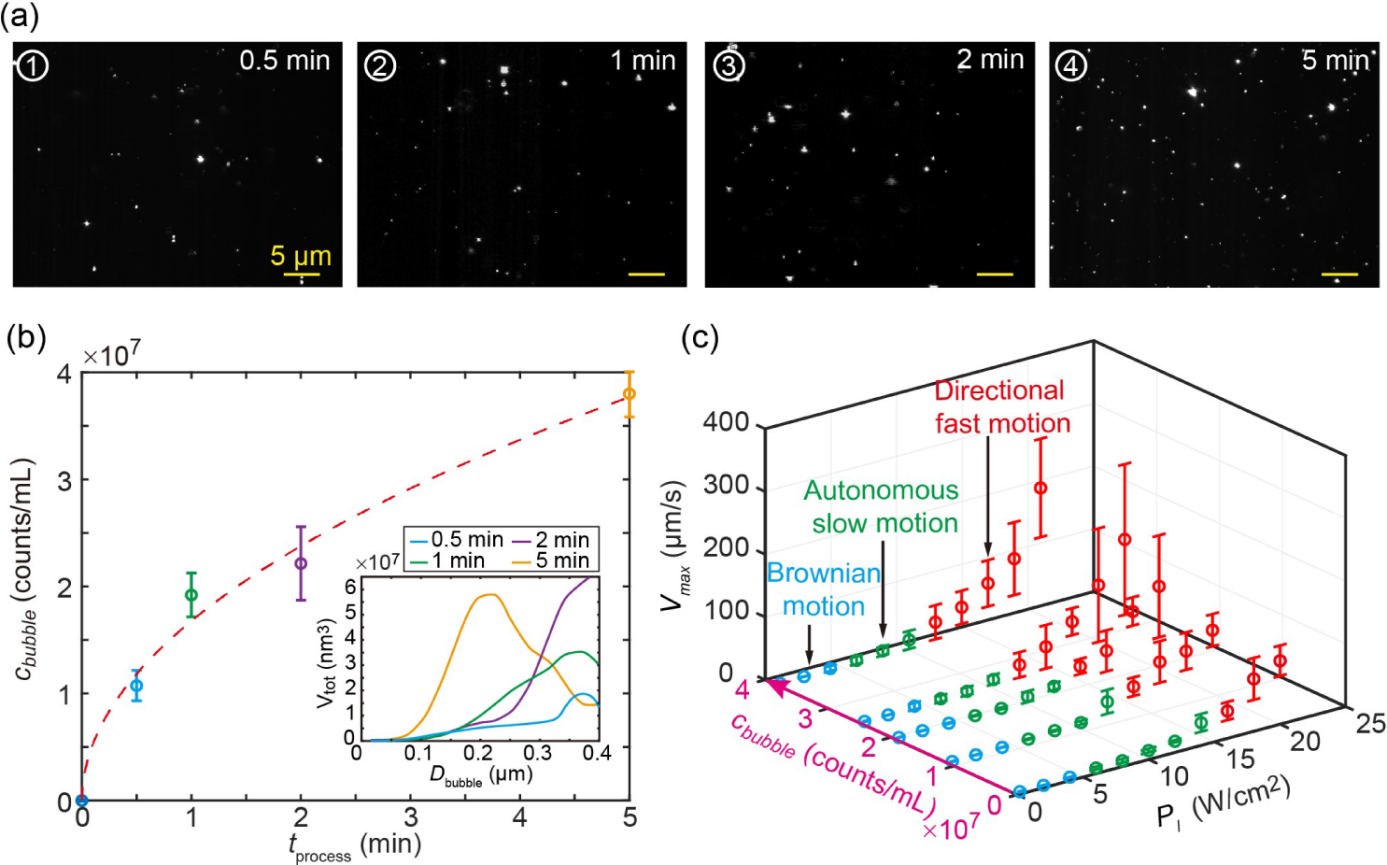
Nanobubbles promoted directional propulsion of CMBs. (a) Dynamic light scattering (DLS) images of nanobubbles in water under ultrasonic with 0.5, 1, 2, and 5 min. (b) Volume distribution of bulk nanobubbles for ultrasonic processing time of 0.5, 1, 2, and 5 min. The dashed curve was drawn to guide the eye. (c) Maximum velocity and phase diagram of CMBs under various nanobubble concentrations and average laser intensities.

To optimize the application of photothermal cavitation bubbles in biomedical fields, it is essential to reduce the required light intensity. Previous studies have demonstrated that contaminants or gas molecules present in water can effectively lower the nucleation temperature of cavitation bubbles.^47,48^ Here, we employed a robust ultrasonic process to incorporate nanobubbles into Triton X-100-infused water, aiming to enhance the efficiency of cavitation bubble generation, thereby facilitating the propulsion of CMBs. Figure 5a displays the dynamic light scattering (DLS) images of nanobubbles at various ultrasonic processing times. As the processing time increased from 0 to 5 min, more nanobubbles formed in the water, with the absolute amounts of nanobubbles per volume increasing from 0 to approximately 4 × 10^7^ counts mL^–1^ (Figure 5b). Simultaneously, the average diameter of the nanobubbles decreased from 400 to 200 nm (Figure 5b inset).

Subsequently, a series of systematic experiments were conducted by varying the scanning laser intensity and nanobubble concentrations while maintaining a frame rate of 7.7 Hz. In the phase diagram depicted in Figure 5c, an increase in the nanobubble concentration *c*_bubble_ from 0 to 4 × 10^7^ counts mL^–1^ resulted in a decrease in the minimum laser intensity required for inducing directional motion of CMBs, reducing it from 16 to 12 W cm^–2^. Meanwhile, the increase in *c*_bubble_ leads to a slight increase in the maximum velocity of the CMB. This suggested that nanobubbles presented in water served as nucleation sites, thereby promoting the formation of cavitation bubbles.

### Selective Plasmid Transfection in Cells Based on CMBs

As shown above, in comparison with the continuous laser, the defocused pulsed and scanning lasers are capable of propelling CMBs at relatively low light intensities. Although the defocused pulsed laser can propel CMBs at significantly lower light intensity ( = 0.05 W cm^–2^) than the scanning laser ( = 10 W cm^–2^), the scanning laser offers controllable directional motion of CMBs by adjusting the scanning orientation, thereby enabling precise navigation of CMBs toward target objectives. In addition, the instantaneous release of substantial energy from laser-induced cavitation bubbles offers a promising approach for the targeted penetration of biological barriers.

**Figure 6:**
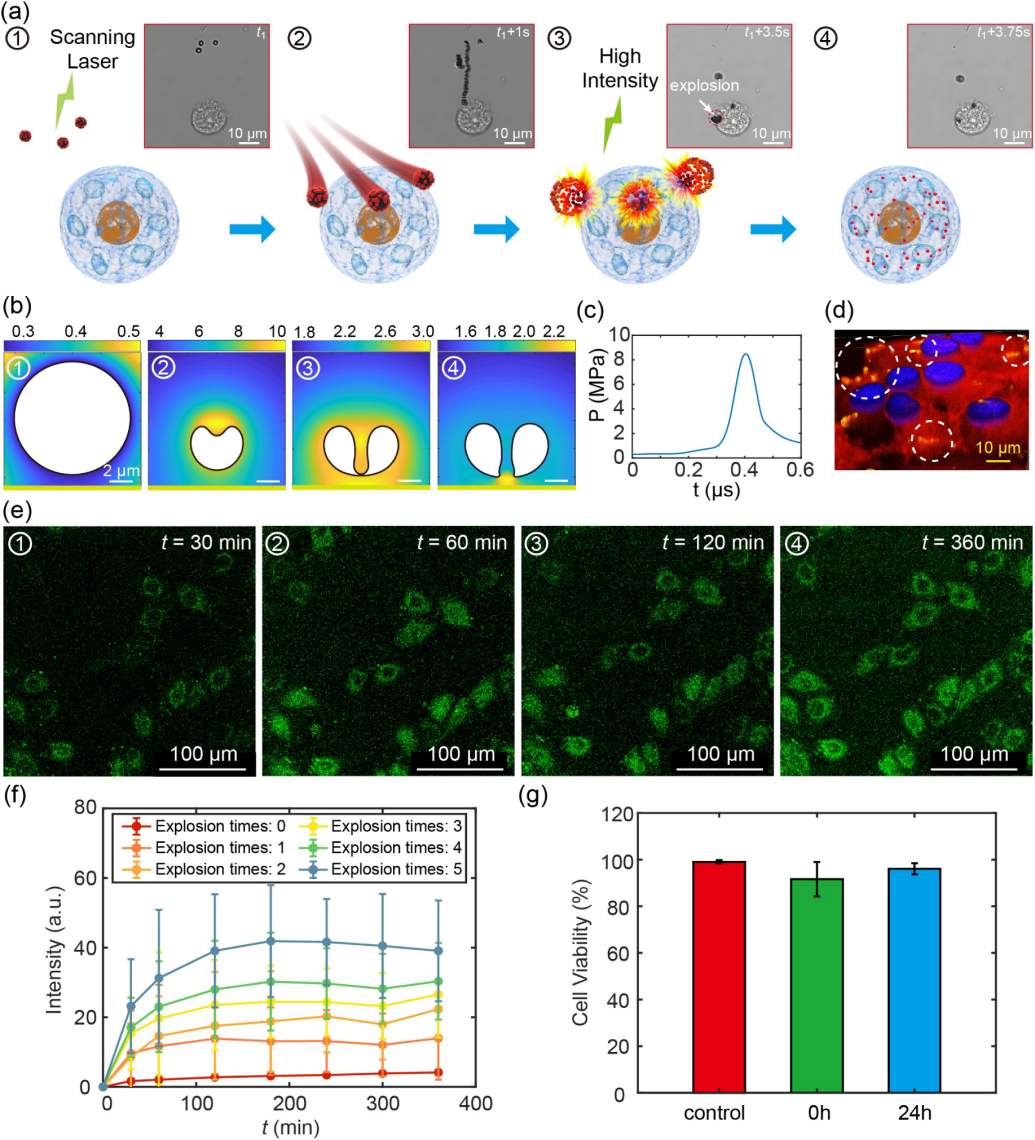
Cell membrane penetration by cavitation bubbles induced by CMBs. (a) Sketches and successive optical images of CMB navigating toward a cell and subsequently penetrating the cell membrane. (b) Numerical simulation of the explosion of a cavitation bubble near a cell membrane. (c) Temporal evolution of the pressure acting on the cell membrane. (d) 3D-constructed fluorescent image of the cell after explosions of CMBs. CMBs, cytoskeleton, and nucleus are labeled by green, red, and blue, respectively. (e) Successive images of green fluorescent protein expression in cells following CMBs’ explosion. (f) Fluorescent intensity expressed by cells as a function of time for various explosion times. (g) Evaluation of cell viability following CMBs’ explosion.

Figure 6a and Movie S10 illustrate the sketch and corresponding recording of the precise navigation and controlled explosion of CMBs near an HeLa cell. Initially, HeLa cells were cultured in DMEM supplemented with CMBs at a concentration of 0.05 mg mL^–1^. Confocal microscopy was utilized to determine the relative positioning of the target cells and CMBs under low light intensity conditions (Figure 6a-1). Subsequently, the scanning orientation was modified and the laser intensity was adjusted to directionally propel the CMBs toward the target cells (Figure 6a-2). Once the CMBs reached the target cells, the light intensity was further increased to induce the explosion of the CMBs (Figure 6a-3), followed by their subsequent collapse (Figure 6a-4).

To validate the efficacy of CMBs’ explosion in penetrating cell membranes, we conducted a numerical simulation of cavitation bubble dynamics near a cell membrane and performed a 3D reconstruction experiment using fluorescent labeling (Supporting Information S2.6). Figure 6b illustrates the pressure contours of a cavitation bubble interacting with a cell membrane, which is depicted as a solid boundary due to the HeLa cell’s viscosity being 10^4^ times higher than water.^51^ Upon reaching its maximum size, the cavitation bubble collapsed, generating a jet flow directed toward the membrane. This resulted in an instantaneous peak pressure intensity of 8 bar (Figure 6c), equivalent to 2 mN, which was 3 orders of magnitude more potent than the force necessary for mechanically opening the cell membrane.^52^ Additionally, a fluorescent labeling experiment was conducted using CMBs labeled in green, the cytoskeleton in red, and the cell nucleus in blue. The 3D confocal image in Figure 6d further indicates that green fluorescent molecules loaded on CMBs could be effectively delivered into cells (We refer to Figure S6 for more details).

Given the ability of CMBs to open cell membranes, we proposed their potential application as a gene delivery tool for the efficient transfection of plasmids into target cells. Plasmids are small extrachromosomal circular DNA molecules (Figure S7), which are commonly introduced into cells through endocytosis. However, this process lacks cellular specificity and is time-consuming, with an average duration of more than 6 h.^53^ Cavitation bubbles could effectively address the aforementioned issues, enabling rapid and targeted plasmid transfection in the cells. As shown in Figure 6e, a series of images capturing the progression of cell fluorescence following the explosion of CMBs loaded with plasmids were illustrated. Over a period of 6 h, there was a gradual increase in the intensity of fluorescence emitted by the cells. In contrast, when the cells were only treated with nonexploded CMBs, only a slight increase in fluorescent intensity was observed after 6 h (Figure S8). Figure 6f depicts the temporal evolution of fluorescent intensities for various explosion times, demonstrating that plasmid expression within cells initiated at 30 min and reached its maximum after approximately 120 min, which was significantly faster than endocytosis-mediated expression.

A cell viability test (Figure 6g) confirmed that nearly 90% of cells remained viable following CMBs’ explosion (Movie S11) both in the short-term (0 h) and long-term (24 h) treatments (Figure S9 and Supporting Information S1.5). Furthermore, we successfully demonstrated the selective targeting of specific cells using CMBs by regulating the scanning region and achieved efficient plasmid transfection, as evidenced by the formation of “HKU” letters through fluorescence imaging (Figure S10). These findings highlight the potential application of CMB-based scanning lasers for precise cell therapy and plasmid transfection.

Although micromotors based on cavitation bubbles have been extensively studied using various techniques, particularly through acoustic fields and focused pulsed lasers,^54^ the scanning laser method introduced in this study offers superior control over micromotor propulsion. This method enables precise directional movement without requiring an additional external field. While focused pulsed lasers can generate cavitation bubbles at specific locations within a fluid, they necessitate ultrahigh light intensities that may damage samples. In contrast, the scanning laser employed in this study operates at a comparatively lower intensity, significantly reducing the risk of sample damage. Moreover, this technique provides a novel approach to modulate the motion direction of micromotors by adjusting the scanning orientation.

## Conclusion

We systematically investigated the motion behaviors of CMBs under three distinct laser conditions: scanning, continuous, and pulsed lasers. When exposed to lasers of sufficient intensity (6 W cm^–2^ for scanning, 3000 W cm^–2^ for continuous, and 0.05 W cm^–2^ for pulsed), CMBs overcame thermal fluctuations and exhibited autonomous motion. Additionally, we observed that the maximum instantaneous velocity of the CMBs could reach 1 m s^–1^ and 100 μm s^–1^ under pulsed and scanning lasers, respectively. We revealed that the nucleation of cavitation bubbles governs the high-speed motion of CMBs. The use of the scanning laser allowed precise manipulation of cavitation bubble nucleation by adjusting the scanning orientation and laser intensity, enabling the accurate directional movement of CMBs. Additionally, considering that gas domains or contamination in a liquid can serve as nucleation sites for bubbles, we introduced nanobubbles into the surrounding liquid to enhance cavitation bubble nucleation, thereby promoting the directional shooting of CMBs.

Additionally, we applied laser-driven CMBs to penetrate cell membranes. By adjusting the scanning orientation, CMBs were successfully guided toward target cells. Subsequently, we strategically increased the laser power, resulting in explosions of CMBs. To demonstrate the efficacy of this explosion-mediated approach in penetrating cell membranes, we conducted a fluorescent labeling experiment and comprehensive simulations. Furthermore, we demonstrated that by loading CMBs with genes, effective cytoplasmic transfection can be achieved.

## Supporting Information Available

Descriptions of materials, setup, experimental procedures, and supporting discussions (PDF)
Movie S1: Autonomous motion of CMBs under a continuous laser with a light intensity of 3000 W cm^–2^ (AVI)
Movie S2: Autonomous motion of CMBs under a pulsed laser with a light intensity of 0.05 W cm^–2^ (AVI)
Movie S3: Autonomous motion of CMBs under a scanning laser with a light intensity of 10 W cm^–2^ (AVI)
Movie S4: Brownian motion of CMBs under background light (AVI)
Movie S5: Directional motion of CMBs under a scanning laser with a light intensity of 20 W cm^–2^ (AVI)
Movie S6: Direction control of CMBs by regulating the scanning orientation (AVI)
Movie S7: Ultrafast motion of a CMB under a pulsed laser captured by a high-speed camera (AVI)
Movie S8: Vapor bubbles driven autonomous motion of CMBs (AVI)
Movie S9: Cavitation bubbles driven directional motion of CMBs under a scanning laser (AVI)
Movie S10: Navigation of CMBs and penetration of cell membrane by CMBs’ explosion (AVI)
Movie S11: Batch explosion of CMBs around HeLa cells (AVI)

## Supplementary Material

ja5c00482_si_001.pdf

ja5c00482_si_002.avi

ja5c00482_si_003.avi

ja5c00482_si_004.avi

ja5c00482_si_005.avi

ja5c00482_si_006.avi

ja5c00482_si_007.avi

ja5c00482_si_008.avi

ja5c00482_si_009.avi

ja5c00482_si_010.avi

ja5c00482_si_011.avi

ja5c00482_si_012.avi
