# Supplementary material for: Photothermal Cavitation-Driven Micromotor to Penetrate Cell Membrane
Source: J Am Chem Soc. 2025 Feb 27;147(10):8906–16. doi: 10.1021/jacs.5c00482 (PMC11912328; doi:10.1021/jacs.5c00482)
Supplement: Supplementary file 1 — ja5c00482_si_001.pdf [file ja5c00482_si_001.pdf]

Supplementary Materials

Photothermal Cavitation-Driven Micromotor to Penetrate Cell Membrane

Binglin Zeng,[†,‡,#] Jialin Lai,[†,#] Jingyuan Chen,[†,⊥,#] Yaxin Huang,[†] Qingxin Guo,[†] Chao Huang,[†] Xiaofeng Li,[†,⊥] Changjin Wu,[¶] Shuai Li,[*,§] and Jinyao Tang[*,†,‡,⊥,//]

†Department of Chemistry, The University of Hong Kong, Hong Kong, China

‡HKU-CAS Joint Laboratory on New Materials and Department of Chemistry, Hong Kong, China

⊥Materials Innovation Institute for Life Sciences and Energy (MILES), HKU-SIRI, Shenzhen, China

¶Department of Mechanical Engineering, The University of Hong Kong, Hong Kong, China

§College of Shipbuilding Engineering, Harbin Engineering University, Harbin, China

// State Key Laboratory of Synthetic Chemistry, The University of Hong Kong, Hong Kong, China

#The authors contributed equally to this work.

E-mail: lishuai@hrbeu.edu.cn; jinyao@hku.hk.

This PDF file includes:

Methods and Materials

Supplementary Discussion

Figs. S1 to S10

Legends for videos S1 to S11

Reference

1 Methods and Materials

1.1 Carbon micro bottles

All relevant reagents including Sodium oleate (SO), 98%, Dribose (98%), furfural (99%), and oleic acid (98%) were supplied by Dickman and used without further purification. The recipe of CMBs were as depicted by ref.[1]. In this procedure, 0.12 mM SO and 0.0075 mM of P123 were first dissolved in 20 mL deionized water, and stirred gently to form a clear solution. Subsequently, 40 mL aqueous solution containing 3 g of ribose was added into the clear solution. After stirring for 30 minutes at room temperature, the mixture became nearly transparent. Then the solution was transferred to an autoclave (approximately 85 mL) and subjected to hydrothermal treatment at 160 ˚C for 12 hours. Upon cooling to room temperature, the solid products were collected by centrifugation (9500 rpm for 15 minutes), washed three times with deionized water, and dried at 70 ˚C overnight. The obtained micro bottle structure was characterized by scanning electron microscopy (SEM, MAIA3 Tescan) and Transmission electron microscope (TEM, Thermo Scientific Talos F200X), respectively. As the SEM and TEM images shown in the inset Figure 1a in the maintext, the diameter of the CMB swimmer is approximately 2 μm.

The photo-thermal properties of synthesized CMBs were evaluated by measuring the temperature increase of aqueous suspensions containing various concentrations of CMBs (1.6, 0.8, 0.4, 0.2 mg mL^{-1}) under a continuous irradiation (532 nm, 2 W cm^{-2}). As shown in Figure S1, such small amounts of CMBs resulted in a temperature increase of 0.5 mL samples from 20 ^oC to over 40 ^oC for all solutions, demonstrating a good photothermal conversion efficiency of CMBs.

1.2 Nanobubble water

To increase the gas concentration in water, Triton X-100 (99%, Sigma) was added into Distilled water with concentration of 0.5 wt%, followed by processing in an ultrasonic cell pulverizer (+UXI) with the power of 80% for 0.5, 1, 2, and 5 min. Subsequently, the resulting gas nanobubbles in the water were characterized using a Dynamic Light Scattering (DLS, PMX120 ZetaView) device.

1.3 HeLa cells

The Hela cell line was obtained from the American Type Culture Collection (ATCC). The cells were cultured at 37 ˚C in 5% CO_2 and maintained in Dulbecco's modified Eagle's medium (DMEM)

supplemented with 10% fetal bovine serum (FBS), 100 μg mL^{-1} penicillin, and 100 μg mL^{-1} streptomycin. Cells were passaged twice a week using trypsin-EDTA solution (all from Invitrogen).

1.4 Setup

The CMBs were exposed to laser irradiation using three different types, including scanning laser, continuous laser and defocused pulsed laser (Fig. 1b in the main text). The scanning laser is generated by a confocal microscope (Leica SP8X) and actuated by an acousto-optic deflector, specifically a TeO_2 crystal. By applying a mechanical wave, periodic density variations within the crystal are induced, which in turn modulate the refractive index. Consequently, the acoustic wave creates a refractive index grating that modulates the incident light into a scanning laser beam. As sketched in in Fig. 1a in the main text, laser irradiation is from the top and the optical images are captured by an optical camera with a 63× objective from the bottom of the CMBs sample. The CMBs are immersed in an aqueous phase and held within a glass tube sealed at both ends with wax. In a typical experiment, all four laser beams with wavelengths of 488, 532, 762 and 1064 nm were switched on for driving CMBs and optical imaging was captured in a scanning area of scanning area is 82×82 μm^2. For the setup with the continuous laser and the pulsed laser, a high power continuous laser source (SC-PRO, YSL Photonics) was equipped with an inverted microscope. Conversely, for the pulsed laser experiment, a pulsed laser generator (Spectra Physics Quanta Ray 532 nm) with the repetition frequency of 10 Hz and the pulse width of 6 ns was employed alongside an upright microscope for imaging. In addition, a high speed camera (VEO 610, Phantom) was employed to capture the dynamic behavior of CMBs under pulsed laser irradiation with the frame rate of 77 kHz. Throughout all experiments, a laser power meter (Sper Scientific 840011) was used to measure the laser intensity.

1.5 Assessment of cell viability following CMBs' explosion

Hela cells were cultured overnight and subsequently washed with PBS three times. Next, cells were exposed to fresh medium containing CMBs at a concentration of 0.05 mg mL^{-1}. After treatment of CMBs' explosion under a scanning laser for around 15 s (Movie S11), the cells were cultured for specific durations (0h or 24h) and stained with 1 μM fluorescence diacetate (FDA) and propidium iodide (PI). Subsequently, cell viability was determined by assessing the red fluorescence signal in correlation with the green fluorescence signal.

1.6 Plasmid transfection experiment

The enhanced green fluorescent protein (EGFP) plasmid was employed to investigate cell transfection by CMBs. Prior to the experiment, Hela cells were cultured until they reached 80% confluency. Subsequently, they were seeded onto a glass-bottom dish and incubated overnight. Following this, DMEM medium was prepared to support the cultured cells. Triton X-100 was added in the DMEM medium with the concentration of 0.5%, and subjected to ultrasound for 5 minutes before being supplemented with CNBs and EGFP. Afterward, the cultured cells were washed three times with phosphate-buffered saline (PBS, Invitrogen), followed by immersion in the prepared DMEM medium. CMBs at a concentration of 0.05 mg mL^{-1} were subsequently added to the DMEM medium. Then the expression of EGFP in Hela cells driven by CMBs was visualized using a confocal laser scanning microscopy.

1.7 Numerical model of interactions between a cavitation bubble and a CMB or a cell membrane

We endeavored to simulate the dynamics of cavitation bubbles under diverse boundary conditions using a Boundary Integral Method (BIM) [2, 3]. Herein, we provide a concise overview of this methodology. Firstly, we estimate the associated Reynolds number (Re), defined as Re = UR_m/v, where U represents the characteristic velocity, R_m signifies the maximum radius the bubble, and v denotes the kinematic viscosity of the fluid. For micron-sized bubbles, Re falls within the range of O(10-10^2). Additionally, we calculate the Weber number (We), defined as We = $\rho U^2 R_m/\sigma$, where ρ signifies the fluid density and σ represents the coefficient of surface tension. Typically, We ranges between O(1-10). Consequently, we employ a combination of viscous potential flow theory [4] and BIM to simulate the dynamic behaviors of cavitation bubbles. The flow field surrounding the bubble is governed by the Laplace equation

$$\nabla^2\varphi = 0, \tag{1}$$

where φ represents the velocity potential, and the velocity field u can be expressed as the gradient of φ, i.e., $u = \nabla\varphi$. Instead of directly solving the Laplace equation, we resolve the boundary integral equation in numerical simulation. It is widely acknowledged that BIM reduces the dimension by one; only the boundaries of the flow field need to be considered and meshed into grids. For the

sake of simplicity, we opt for the axisymmetric BIM in this investigation owing to the geometric characteristics involved.

The influence of viscosity within the boundary layers can be addressed through dynamic boundary conditions applied to the bubble surface, expressed as follows:

$$\frac{\mathrm{d}\varphi}{\mathrm{d}t} = \frac{P_\infty - P_b}{\rho} + \frac{1}{2}|\nabla\varphi|^2 + \frac{\sigma\kappa}{\rho} - 2v\frac{\partial^2\varphi}{\partial n^2}, \tag{2}$$

where P_∞ is the ambient hydrostatic pressure, P_b the gas pressure inside the bubble, ρ the density of the fluid. The last two terms on the right side present the surface tension and viscous corrections, respectively. This comprehensive boundary condition is crucial for accurately simulating the behavior of cavitation bubbles in viscous fluids and is incorporated into the computational model to capture the complex dynamics of bubble formation, growth, and collapse under various conditions. The gas pressure inside the bubble is calculated from the ideal gas equation. To update the velocity potential φ on the bubble surface in the time domain, Equation 2 is used, and numerical methods such as the fourth-order Runge-Kutta method are employed for enhanced accuracy in the simulation process.

The kinematic boundary conditions on the bubble surface is expressed as:

$$\frac{\mathrm{d}r}{\mathrm{d}t} = \nabla\varphi, \tag{3}$$

which is essential for updating the location of the bubble surface over time, accounting for the movement of the bubble surface itself.

The boundary condition for a wetted particle surface or rigid wall is expressed as follows:

$$\frac{\partial\varphi}{\partial n} = \boldsymbol{U}\cdot\boldsymbol{n}, \tag{4}$$

where U is the velocity of the object (e.g., the translational velocity of the particle), n denotes the outward unit normal vector on the boundary surface. This boundary condition ensures that no fluid penetrates the boundary surface.

The first numerical setup is about the jetting behavior of a cavitation bubble near a cell that attached to the rigid wall. Due to the relatively flat morphology of cells adhering to the basal wall surface, as well as the significantly higher fluid viscosity inside the cells compared to water, we will employ a bubble model near rigid boundaries to assess and analyze the jet velocity and impact

pressure of the bubble. We employ wall Green's functions and the method of images [3] to avoid directly discretizing the wall boundary, thus simulating the dynamic behavior of bubbles near the wall. This approach allows us to conserve significant computational resources.

In the second numerical setup for the bubble-particle interaction, we utilize an auxiliary function method [3] to decouple the calculation of the hydrodynamic force and acceleration driven by the bubble. This approach not only accounts for bubble dynamic behaviors but also considers particle acceleration. As particle motion encounters fluid viscous resistance, we have included this factor in our computational model as well. For the determination of the drag coefficient of particulate matter, interested readers can refer to Borkent's work [5]. More details about the numerical models can be referenced from our previous research.

2 Supplementary Discussion

2.1 Particle tracking

The locomotive trajectories of micro motors were tracked and processed by a homemade MATLAB codes based on the binarization algorithm.

2.2 Instantons laser power calculation for the scanning laser

The average laser intensities were measured by a laser power meter. To obtain the instantaneous laser intensity deposited on the micro motors, we need to calculate the laser intensity for each scanning point, which is given by

$$P_{point} = \frac{E_{point}}{S_{point} t_{point}}, \tag{5}$$

where E_{point}, S_{point} and t_{point} are the deposited energy, area and dwell time for each scanning point. The dwell time t_{point} can be given by

$$t_{point} = \frac{1}{f\, {L_n}^2}, \tag{6}$$

where f is the frame rate for confocal imaging and L_n is the line number, namely the number of points along one scanning line. Note that the average laser intensity for total scanning area is given by the accumulation of each point, then times the frame rate f and divided the scanning area,

$$P_l = \frac{E_{point} {L_n}^2 f}{S_{scan}}, \tag{7}$$

By combining Eqs. (5) to (7), we get that

$$P_{point} = \frac{S_{scan}}{S_{point}} P_l. \quad (8)$$

It should be noted that the deposition of laser energy onto an object occurs only when the laser scans it. Therefore, it is essential to consider the ratio between the projected area of the scanning point and the object. The instantaneous laser intensity deposited on a micro bottle can be determined as follow:

$$P_{bottle} = \frac{S_{point}}{S_{bottle}} P_{point} = \frac{S_{scan}}{S_{bottle}} P_l. \quad (9)$$

When the line number of scanning is L_n = 256, the ratio of the projected area of a micro bottle to the total scanning area, S_{scan}/S_{bottle} is approximately 1300. Considering an example with laser intensity of 20 W cm^{-2}, we estimated the instantaneous laser intensity deposited on a micro bottle $P_{bottle} \approx$ 26000 W cm^{-2}.

2.3 Estimation of thermophoretic effect on CMBs' propulsion

The thermophoretic effect has been widely employed to drive micro-motors, especially through the mechanism of thermophoresis, which induces a temperature gradient across asymmetrically structured micro-motors. However, thermophoresis alone cannot fully explain the ultra-fast and directional motion observed in our micro-bottles. Theoretical and experimental evidence is provided below.

In theory, for a thermophoresis propelled micro motor system, the velocity of a micromotor is given by:

$$V = -DS_T \nabla T, \quad (10)$$

where D is the diffusion coefficient of micro motors in water, S_T is the Soret coefficient and ∇T is the temperature gradient across a micromotor. By fitting the MSD data of micro motors' Brownian motion, we can extract $D \approx$ 0.23 μm^2/s from MSD = 4Dt (Figure S2a). For a colloidal particle interacting with a pure and incompressible solution, the Soret coefficient should be proportional to the temperature derivative of the product of the interfacial tension σ_{ab} and a characteristic length l [6], following that

$$S_T = \frac{4\pi R}{k_B T} \frac{\partial (l \sigma_{ab})}{\partial T}, \quad (11)$$

where R is particle radius, k_B is Boltzmann constant, T is temperature, and the characteristic length l is related to the width of the attached fluid layer near the particle. With k_B =1.380649 $\times 10^{-23}$J K^{-1}, T = 300 K, R = 1 μm, and l typically set to 0.1 to 0.2 nm [7], the value of $\partial\sigma_{ab}/\partial T$ is 0.04×10^{-3} N m^{-1} K^{-1}(Figure S2b) [8]. Consequently, we obtain $S_T \approx$ 12 to 24 K^{-1}, which aligns with previously reported values for Janus micro-motors driven by thermophoresis [9].

According to Eq. 10, to estimate the propulsion velocity of micro motors due to thermophoresis, it is essential to determine the temperature gradient across the motor. As illustrated in Figure S3a, we numerically simulated the temperature field surrounding a micro bottle under uniform laser irradiation at an intensity of 3000 W cm^{-2}. Figure S3b depicts the temperature evolution near the head (T_{head}) and the open end (T_{open}) of the micro bottle, revealing a temperature difference between T_{head} and T_{open} attributed to the asymmetry of the micro bottle. Figure S3c presents the average temperature gradient ∇T across the micro bottle, indicating that as T_{head} increases from 20 to 30 ℃, ∇T rises from 0 to 0.8 K μm^{-1} and remains constant thereafter, which is similar to the typical temperature gradient in the micro motors system driven by thermophoresis [9-11]. Subsequently, substituting ∇T = 0.8 K μm^{-1}, S_T = 24 K^{-1}, and D = 0.23 μm^2 s^{-1} into Eq. 5 yields $V \approx$ 4.4 μm s^{-1}. This value aligns closely with the experimentally observed propulsion velocity of approximately 10 μm s^{-1} during autonomous motion of micro bottles (Figures 1c-2 and 1e-2 in the main text), suggesting that thermophoresis may contribute to the autonomous motion of micro bottles (marked by green in Figure 2d in the main text).

However, thermophoresis effect can not explain the ultra-fast and unified directional motion of micro bottles under the scanning laser (3rd phase in Figure 2d in the main text), which is the core in this study. During the directional motion process, micro bottles exhibit the instantaneous velocity of around 130 μm s^{-1} (Figure 2a-3). Substituting $D \approx$ 0.23 μm^2/s, $S_T \approx$ 24 K^{-1} and V = 130 μm s^{-1} into Eq. 5, we obtained that ∇T across the micro-motor must reach approximately 23 K μm^{-1}, which is far beyond the ∇T value of around 0.8 K μm^{-1} resulting from photo thermal effect. Beyond that, if we consider the propulsion velocity of 1 m s^{-1} for the pulsed laser (Figure 2a-2 in the main text), ∇T must reach 3.6×10^5 K μm^{-1}. Therefore, we conclude that thermophoresis cannot generate such ultra-fast propulsion for micro-motors.

In addition, thermophoresis cannot induce unified directional propulsion of the micro bottles, as shown in Figure 1e-3 in the main text and Movie S6. Since the asymmetric structure of the micro bottle arises from its open end, the temperature gradient across the motor is theoretically aligned along the axis from the head to the open end. Consequently, self-propulsion force resulting from thermophoresis would be parallel to this axis. Therefore, given that the micro bottles are randomly dispersed in water, thermophoresis would cause each micro bottle to move along its individual axis, rather than producing a unified directional motion.

2.4 Velocity calculation of a carbon micro bottle propelled by a water jet

Since the Peclet number (the ratio of thermal convection and thermal diffusion) Pe = $Rv_b/\alpha \sim 10^{-4}$ for a CMB with autonomous motion, where $R \approx 1$ μm is the radius of the CMB, $v_b \approx 10$ μm s^{-1} is the propelling velocity of the CMB and $\alpha = 0.143 \times 10^{-6}$ m^2 s^{-1} is the thermal diffusion coefficient in water. Therefore, the thermal induced temperature field can be considered as quasistationary, and the influence of the motion of CMB on the temperature field can be neglected. Here we used COMSOL Multiphysics (Version 5.4) to numerically estimate the temperature field and the induced flow field around a CMB. The evolution of the temperature, pressure and velocity field of water surrounding the CMB under a boundary heat source with an intensity of 3000 W cm^{-2} is illustrated in Figure S4. The thermal-induced volume expansion of water inside the CMB leads to the generation of a jet through its neck. Figure S5c presents the temporal evolution of jet velocity, demonstrating that within 1 μs, the jet velocity increases to 18 mm s^{-1}. This high-speed jet lasts for approximately 2 μs before declining to zero within 400 μs. This thermal induced water jet can generate an opposite propulsion on the CMB. According to the momentum theorem, the instantaneous velocity v_b of the CMB can be calculated by

$$m_b v_b = \int_{t_1}^{t_1+\Delta t} \rho_w S_{neck} v_{jet}^2 dt \, , \tag{12}$$

where m_b is the mass of a CMB, ρ_w is the density of water, S_{neck} is the sectional area of the neck of a CMB and v_{jet} is the water jet velocity. By substituting the value of $m_b \approx 10^{-14}$ kg, $\rho_w = 1000$ kg m^{-3}, $S_{neck} \approx 2 \times 10^{-13}$ m^2 and the value of the jet velocity in Figure S5c, we obtain the propulsion velocity v_b of the CMB. As shown in Figure S5d, we find that the water jet can generate an unsustainable highest propulsion velocity for a CMB of approximately 10 μm s^{-1} for 2 μs, indicating that the water jet is not enough to maintain a continuous automatous motion of CMB.

2.5 Numerical simulation of temperature field under a scanning laser

The temperature distribution during laser scanning through the upper half of a micro bottle was simulated using COMSOL Multiphysics (Version 5.4). In this model, a two-dimensional axisymmetric structure of a micro bottle was immersed in water. The bottle was positioned in a fixed location, and the surrounding water was assumed to be incompressible. A boundary heat source with a series of flux values of 26000, 20800, 15600, 10400, 5200 and 260 W cm^{-2} corresponding to average laser intensities of 20, 16, 12, 8, 4 and 0.2 W cm^{-2} were applied using heat transfer module. An emissivity efficiency of approximately 0.8, commonly employed for carbon materials, was utilized to simulate the conversion efficiency from light to heat.

2.6 Fluorescent labeling test for overcoming membrane barriers

A fluorescent labeling test was performed to demonstrate the ability of cavitation bubbles induced by CMBs to overcome membrane barriers of Hela cells. In this demonstration, the CMBs were labeled by green fluorescent protein (GFP, 99%, Sigma-Aldrich) in green. Cytoskeleton were labeled by filaggrin (99%, Sigma-Aldrich) in red and cell nucleus were labeled by 4',6-diamidino-2-phenylindole (DAPI, 99%, Sigma-Aldrich) in blue. A confocal microscope was employed for the three-dimensional reconstruction of Hela cells subsequent to CMBs detonation. Laser beams with wavelengths of 488, 532, and 762 nm were employed to excite DAPI, GFP, and filaggrin correspondingly.

3 Supplementary Figures

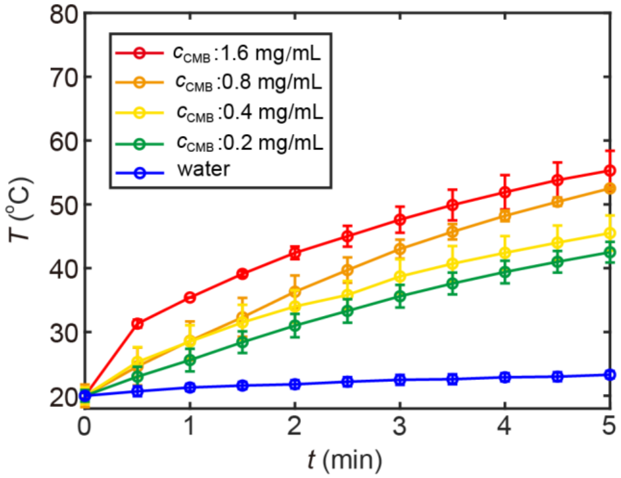

Figure S1. Temporal evolution of temperature in 0.5 mL aqueous solution containing CMBs at concentrations of 1.6, 0.8, 0.4 and 0.2 mg/mL under continuous laser irradiation with a light intensity of 1 W cm^{-2}. Distilled water was used as the control group.

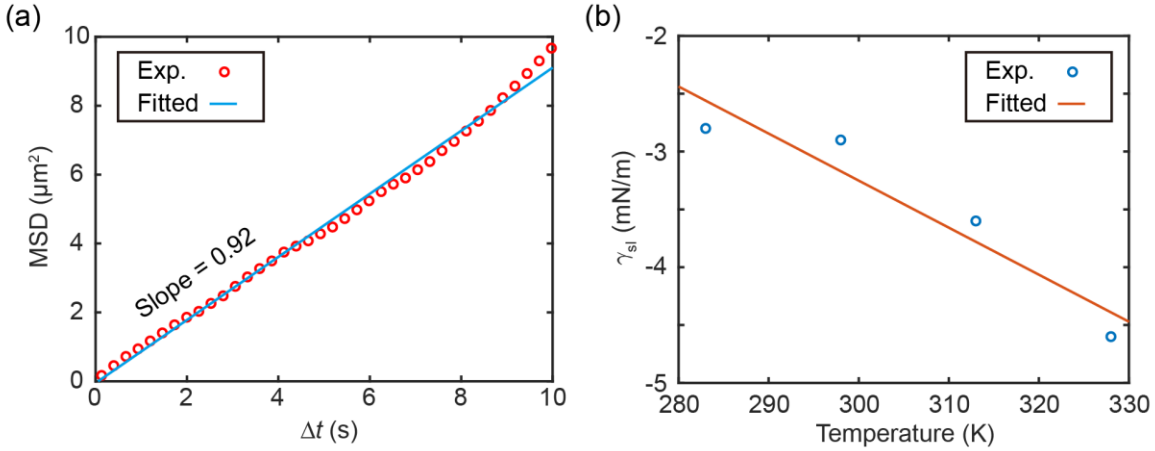

Figure S2. (a) MSD of a micro bottle with Brownian motion. (b) Interfacial tension of water and carbon as a function of temperature, redrawn from Fereshte et. al [8].

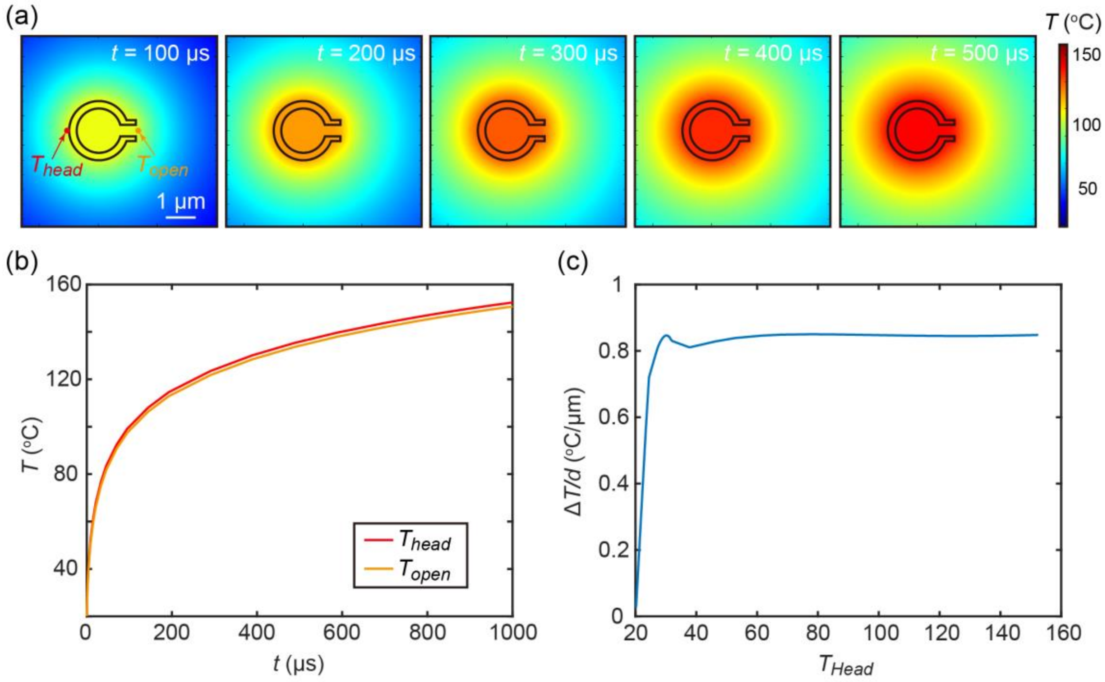

Figure S3. (a) Numerical temperature field around a micro bottle under a continuous laser at the light intensity of 3000 W cm^{-2}. (b) Temporal evolution of temperature near the head T_{head} and the open end T_{open} of a micro bottle. (c) Average temperature gradient around the micro bottle $\nabla T \approx \Delta T/d$ as a function of T_{head}, where $\Delta T = T_{head} - T_{open}$ and d is the diameter of the micro bottle.

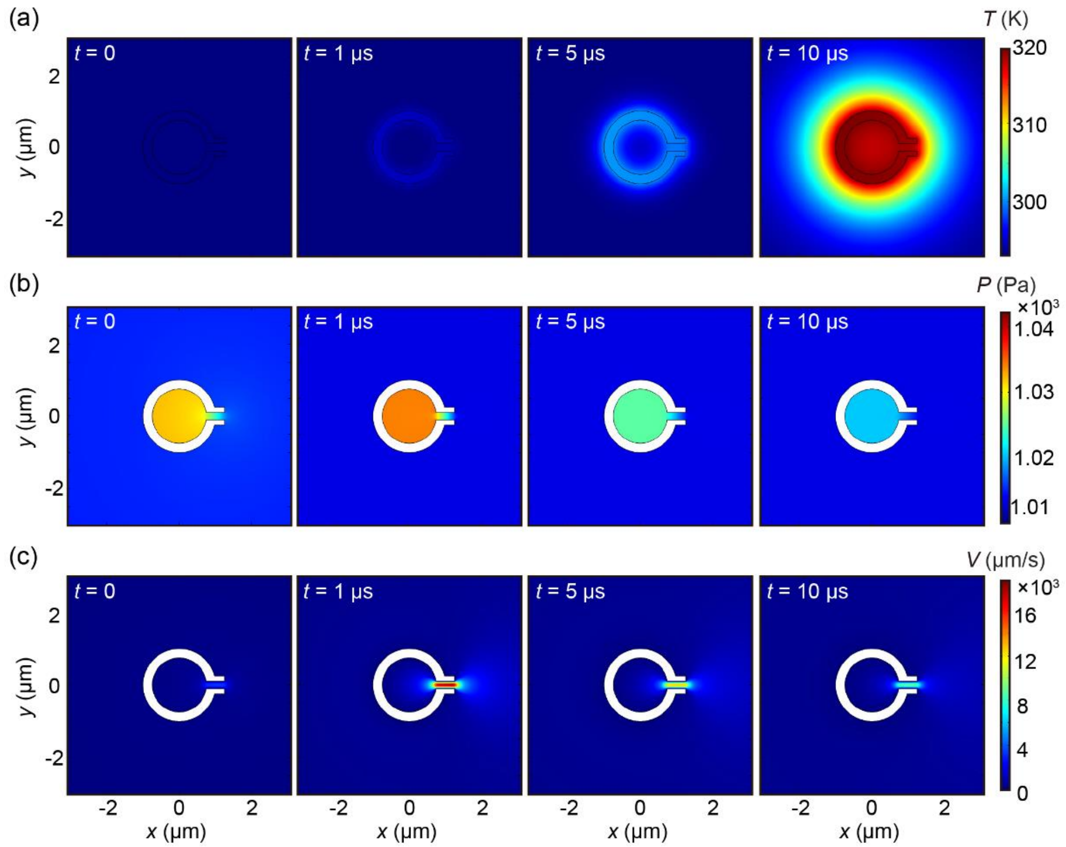

Figure S4. Numerical results of the temperature field (a), pressure field (b) and the velocity field (c) near a CMB under the boundary heat source of 3000W cm^{-2}.

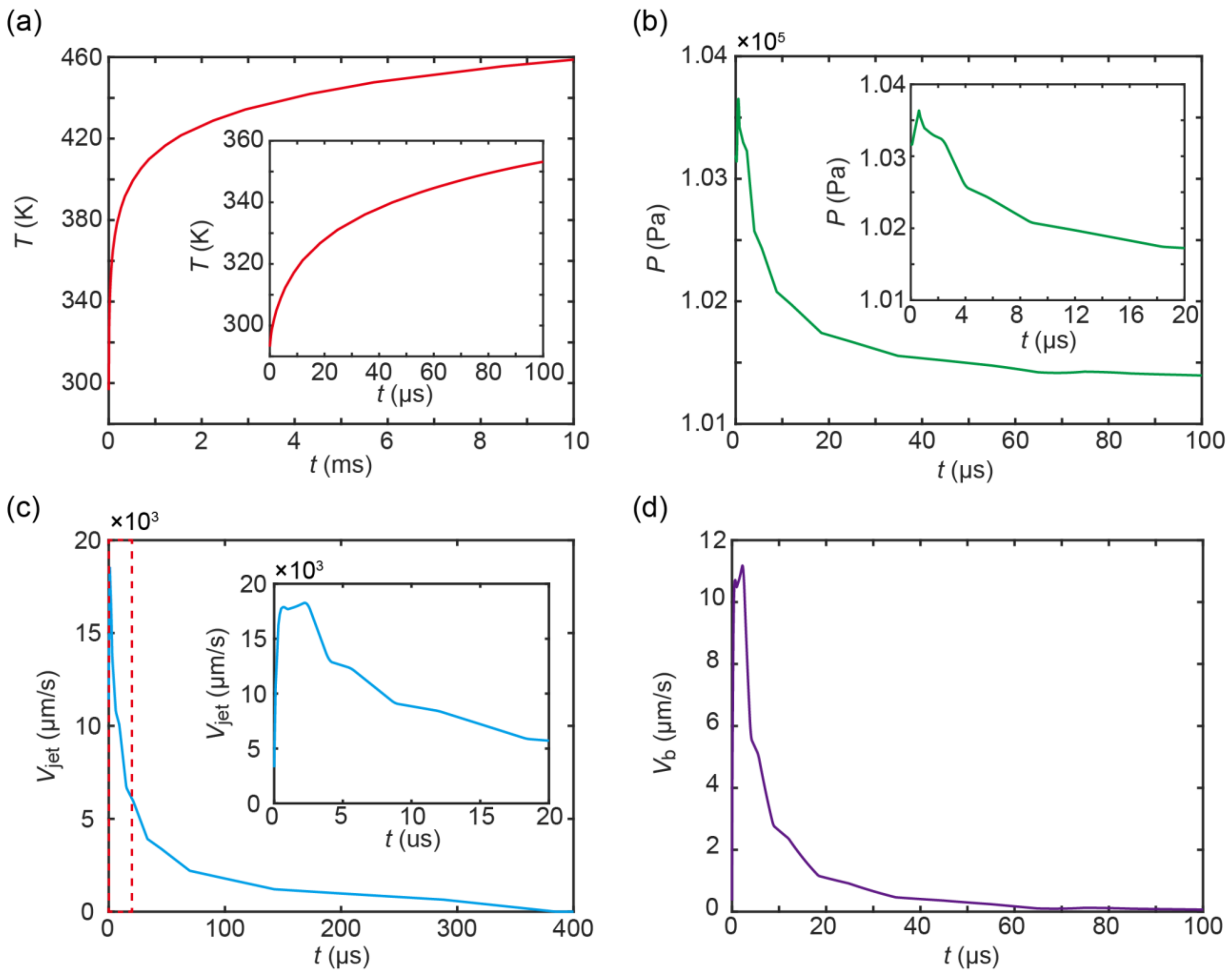

Figure S5. Temporal evolution of the temperature (a) and the pressure (b) inside a CMB, the jet velocity through the neck of the CMB (c) and the calculated propelling velocity (d) of a CMB.

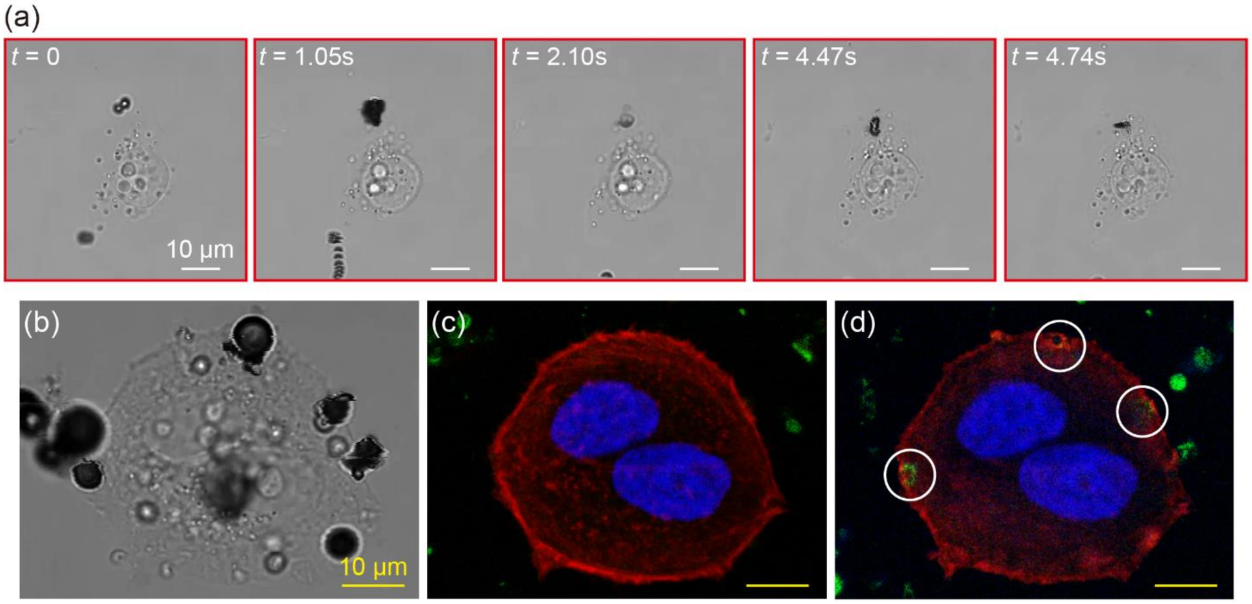

Figure S6. (a) Successive optical images of a micro bottle explosion near an adherent Hela cancer cell. (b) Optical image of multiple bubbles explosion around a Hela cell. (c, d) In situ fluorescent images of a Hela cell before and after explosion of CMBs. Cytoskeleton was labeled by red, cell nucleus was labeled by blue and CMBs were labeled by green.

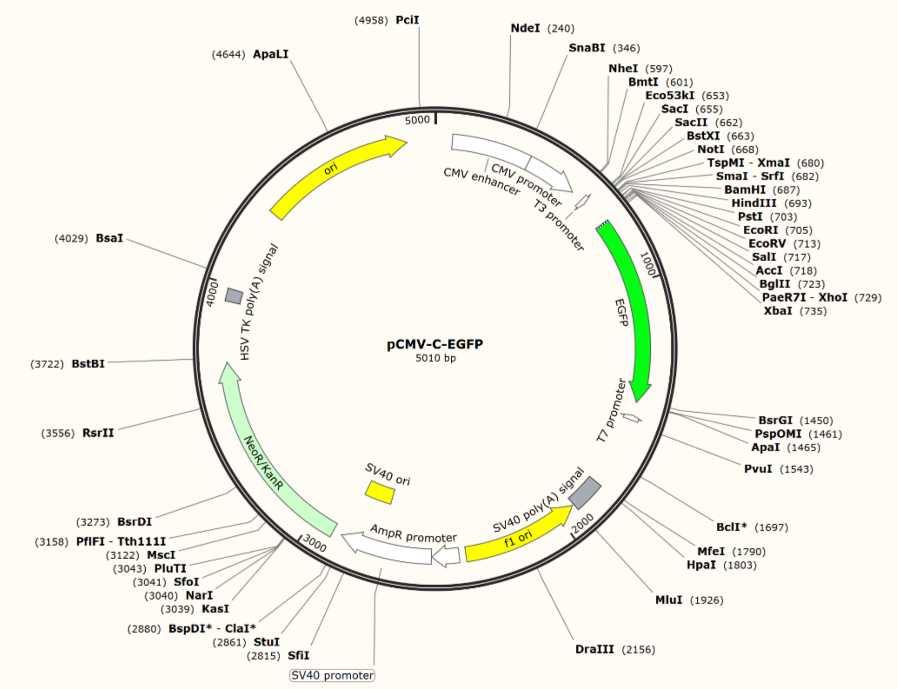

Figure S7. Plasmid sequence map.

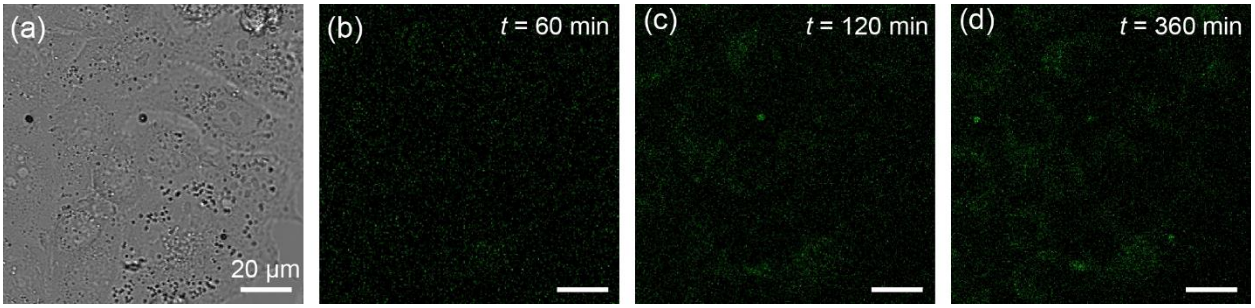

Figure S8. (a) Optical image of Hela cells. (b-d) Successive fluorescent images of green fluorescent protein expression in Hela cells treated by CMBs without explosion.

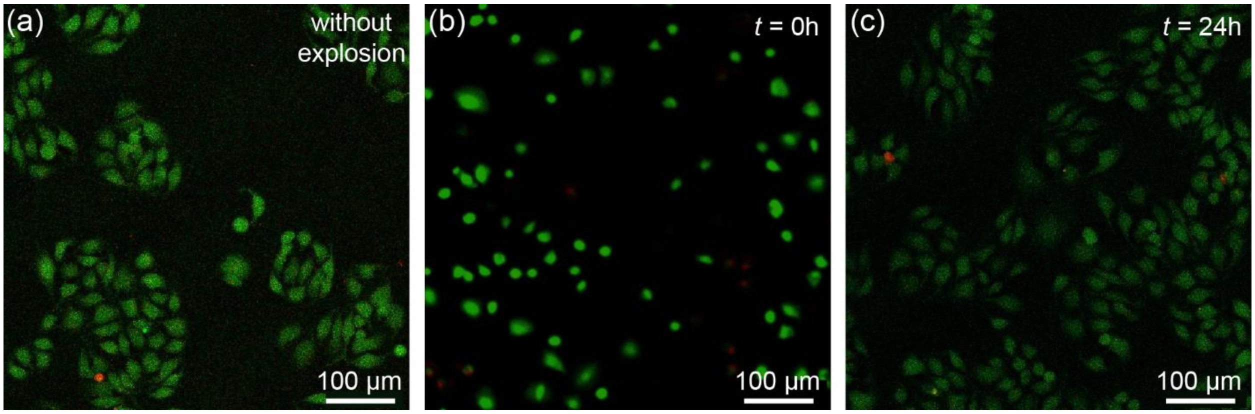

Figure S9. Representative fluorescent images of Hela cells before (a) and following CMBs' explosion in the short (b) and long term (c). Active and dead cells were labeled by green and red, respectively.

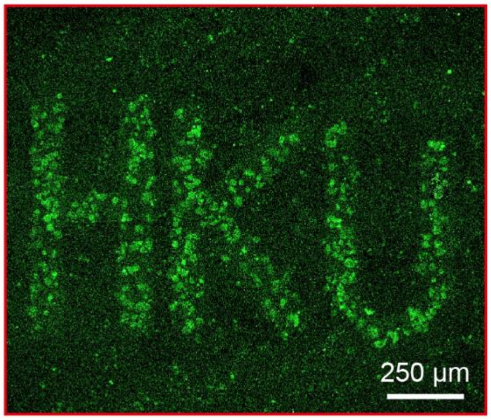

Figure S10. 'HKU' pattern of cells formation by regulating scanning region of scanning laser.

4 Description of Movies

Movie S1: Autonomous motion of CMBs under a continuous laser with the light intensity of 3000 W cm^{-2}.

Movie S2: Autonomous motion of CMBs under a pulsed laser with the light intensity of 0.05 W cm^{-2}.

Movie S3: Autonomous motion of CMBs under a scanning laser with the light intensity of 10 W cm^{-2}.

Movie S4: Brownian motion of CMBs under background light.

Movie S5: Directional motion of CMBs under a scanning laser with the light intensity of 20 W cm^{-2}.

Movie S6: Direction control of CMBs by regulating the scanning orientation.

Movie S7: Ultrafast motion of a CMB under a pulsed laser captured by a high-speed camera.

Movie S8: Vapor bubbles driven autonomous motion of CMBs.

Movie S9: Cavitation bubbles driven directional motion of CMBs under a scanning laser.

Movie S10: Navigation of CMBs and penetration of cell membrane by CMBs' explosion.

Movie S11: Batch explosion of CMBs around Hela cells.

References:

[1] Chen, C., Wang, H., Han, C., Deng, J., Wang, J., Li, M., Tang, M., Jin, H., Wang, Y.: Asymmetric flasklike hollow carbonaceous nanoparticles fabricated by the synergistic interaction between soft template and biomass. J. Am. Chem. Soc. **139**(7), 2657–2663 (2017)

[2] Han, R., Zhang, A.-M., Tan, S., Li, S.: Interaction of cavitation bubbles with the interface of two immiscible fluids on multiple time scales. J. Fluid Mech. **932**, 8 (2022)

[3] Li, S., Zhang, A.-M., Han, R., Ma, Q.: 3d full coupling model for strong interaction between a pulsating bubble and a movable sphere. J. Comput. Phys. **392**, 713–731 (2019)

[4] Joseph, D.D., Wang, J.: The dissipation approximation and viscous potential flow. J. Fluid Mech. **505**, 365–377 (2004)

[5] Borkent, B.M., Arora, M., Ohl, C.-D., De Jong, N., Versluis, M., Lohse, D., Mørch, K.A., Klaseboer, E., Khoo, B.C.: The acceleration of solid particles subjected to cavitation nucleation. J. Fluid Mech. **610**, 157–182 (2008)

[6] Parola A, Piazza R. Particle thermophoresis in liquids[J]. Eur. Phys. J. E, 2004, 15(3): 255-263.

[7] Piazza R, Parola A. Thermophoresis in colloidal suspensions[J]. J. Phys.: Condens. Matter, 2008, 20(15): 153102.

[8] Taherian F, Marcon V, van der Vegt N F A, et al. What is the contact angle of water on graphene?[J]. Langmuir, 2013, 29(5): 1457-1465.

[9] Jiang H R, Yoshinaga N, Sano M. Active motion of a Janus particle by self-thermophoresis in a defocused laser beam[J]. Phys. Rev. Lett., 2010, 105(26): 268302.

[10] Dai J, Cheng X, Li X, et al. Solution-synthesized multifunctional janus nanotree microswimmer[J]. Adv. Funct. Mater., 2021, 31(48): 2106204.

[11] Wang J, Wu H, Zhu X, et al. Ultrafast light-activated polymeric nanomotors[J]. Nat. Commun., 2024, 15(1): 4878.
